# *Streptomyces lividans* 66 produces a protease inhibitor via a tRNA-utilizing enzyme interacting with a C-minus NRPS

**DOI:** 10.1093/jimb/kuad021

**Published:** 2023-09-05

**Authors:** César Aguilar, Karina Verdel-Aranda, Hilda E Ramos-Aboites, Cuauhtémoc Licona-Cassani, Francisco Barona-Gómez

**Affiliations:** Evolution of Metabolic Diversity Laboratory, Unidad de Genómica Avanzada (Langebio), Centro de Investigación y Estudios Avanzados del Instituto Politécnico Nacional (Cinvestav-IPN), Irapuato, Guanajuato, CP 36821, México; Evolution of Metabolic Diversity Laboratory, Unidad de Genómica Avanzada (Langebio), Centro de Investigación y Estudios Avanzados del Instituto Politécnico Nacional (Cinvestav-IPN), Irapuato, Guanajuato, CP 36821, México; Evolution of Metabolic Diversity Laboratory, Unidad de Genómica Avanzada (Langebio), Centro de Investigación y Estudios Avanzados del Instituto Politécnico Nacional (Cinvestav-IPN), Irapuato, Guanajuato, CP 36821, México; Evolution of Metabolic Diversity Laboratory, Unidad de Genómica Avanzada (Langebio), Centro de Investigación y Estudios Avanzados del Instituto Politécnico Nacional (Cinvestav-IPN), Irapuato, Guanajuato, CP 36821, México; Evolution of Metabolic Diversity Laboratory, Unidad de Genómica Avanzada (Langebio), Centro de Investigación y Estudios Avanzados del Instituto Politécnico Nacional (Cinvestav-IPN), Irapuato, Guanajuato, CP 36821, México

**Keywords:** Small peptide aldehyde, Protease inhibitors, L/F transferase, tRUE, Livipeptin, Evolutionary genome mining, *Streptomyces lividans*

## Abstract

Small peptide aldehydes (SPAs) with protease inhibitory activity are naturally occurring compounds shown to be synthesized by non-ribosomal peptide synthetases (NRPS). SPAs are widely used in biotechnology and have been utilized as therapeutic agents. They are also physiologically relevant and have been postulated to regulate the development of their producing microorganisms. Previously, we identified an NRPS-like biosynthetic gene cluster (BGC) in *Streptomyces lividans* 66 that lacked a condensation (C) domain but included a tRNA-utilizing enzyme (tRUE) belonging to the leucyl/phenylalanyl (L/F) transferase family. This system was predicted to direct the synthesis of a novel SPA, which we named livipeptin. Using evolutionary genome mining approaches, here, we confirm the presence of L/F transferase tRUEs within the genomes of diverse *Streptomyces* and related organisms, including fusions with the anticipated C-minus NRPS-like protein. We then demonstrate genetic functional cooperation between the identified L/F-transferase divergent tRUE homolog with the C-minus NRPS, leading to the synthesis of a metabolic fraction with protease inhibitory activity. Semisynthetic assays in the presence of RNAse revealed that the productive interaction between the tRUE and the C-minus NRPS enzymes is indeed tRNA dependent. We expect our findings to boost the discovery of SPAs, as well as the development of protease-mediated biotechnologies, by exploiting the uncovered genetic basis for synthesizing putative acetyl-leu/phe-arginine protease inhibitors. Furthermore, these results will facilitate the purification and structural elucidation of livipeptin, which has proven difficult to chemically characterize.

**Significance:**

The discovery of natural products biosynthetic genes marks a significant advancement in our understanding of these metabolites, for example of their evolution, activity, and biosynthesis, but also opens biotechnological opportunities and knowledge to advance genome mining approaches. We made this possible by uncovering a new biosynthetic pathway in *Streptomyces lividans* 66 shown to direct the synthesis of a strong protease inhibitor, termed livipeptin, following unprecedented biosynthetic rules and genes. Thus, by shedding light on the genetic mechanisms predicted to govern the production of acetyl-leu/phe-arginine protease inhibitors, including the elusive livipeptin, this study enables novel protease-mediated biotechnologies as well as approaches for discovering protease inhibitors from genome data.

## Graphical Abstract

**Graphical Abstract:**
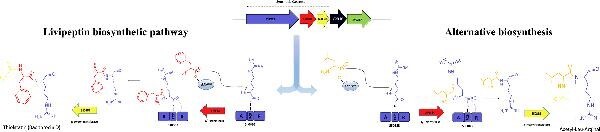
Livipeptin, a novel compound with protease inhibitory properties, has been discovered, along with the identification of the underlying biosynthetic genes, including the remarkable presence of an unprecedented tRNA-utilizing enzyme in *Streptomyces lividans* 66, presenting promising prospects for biotechnological and therapeutic advancements.

## Introduction

Small peptide aldehydes (SPAs) are metabolites with protease inhibitory activity (Mullowney et al., 2018). Their production has been reported in several bacterial species belonging to the phyla Actinobacteria, Cyanobacteria, and Firmicutes, as well as in fungal species belonging to the families *Aspergillaceae* and *Apiosporaceae* (Sabotič & Kos, 2012). SPAs molecular weight ranges between 300 and 900 Da, and they are characterized by N-terminal groups capped with acyl groups or with ureido-amino acid groups, giving rise to acylated or aminoalkyl ends with a terminal carboxylic group, and a terminal aldehyde group derived from the carboxyl-terminal modification of the peptide chain (Bullock et al., 1996). Biosynthetically, SPAs have been reported to be produced in *Streptomyces* by non-ribosomal peptide synthetases (NRPS) with a reductase (R) domain, which mediates the release of the nascent peptide, and at the same time, the formation of the aldehyde group (Chen et al., 2013; Maxson et al., 2016; Mullowney et al., 2018). These reactions lead to the chemical warhead by which SPAs covalently interact with serine or cysteine protease active site residues, giving place to hemiacetals or hemithioacetals, irreversibly inhibiting proteases enzymatic activity (Brayer et al., 1979; Wlodawer et al., 2001).

**Fig. 1.**
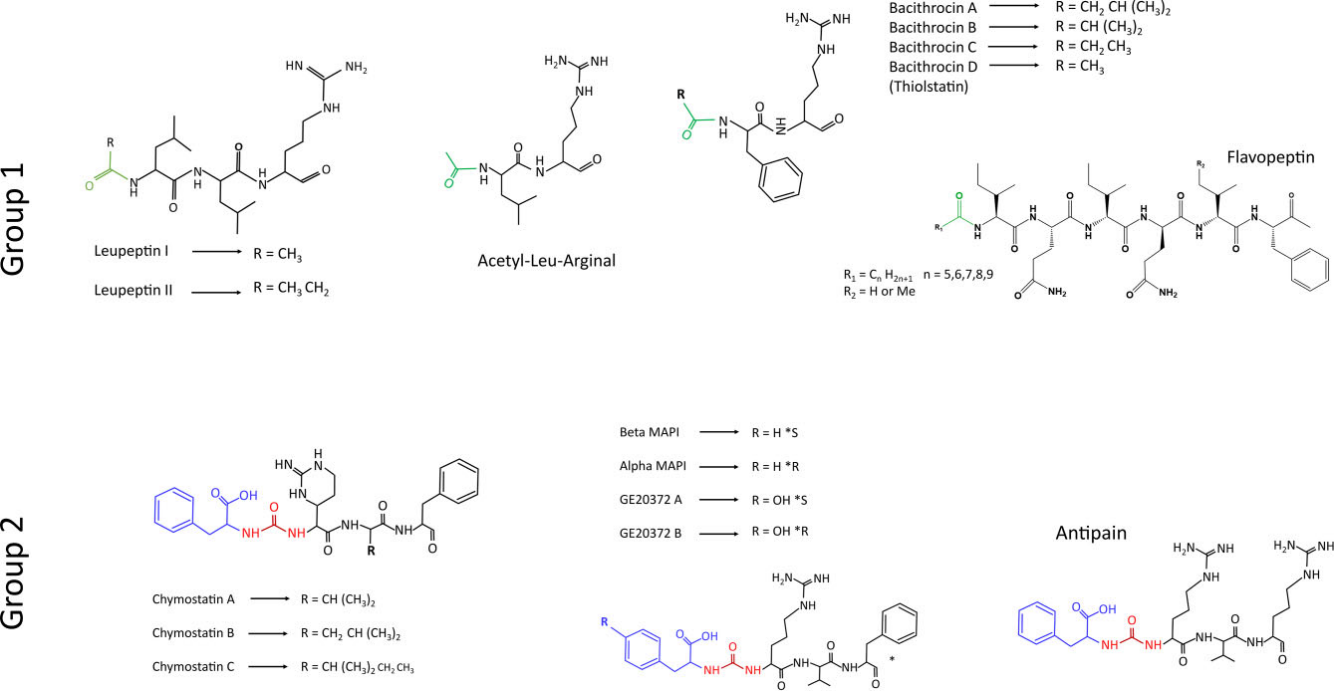
Chemical structures of selected small peptide aldehydes. Structural features of predicted livipeptin (or thiolstatin) and other related SPAs are shown. The characteristic moiety protecting the acetyl group in this family is shown in green.

Based on their functional groups, SPAs can be divided into two groups (Fig. 1): (i) those with a terminal group protected by an acyl group, including acetyl-leu-arginal (Nishikiori et al., 1984), bacithrocins A–D (Kamiyama et al., 1994), flavopeptin (Chen et al., 2013), leupeptin (Aoyagi et al., 1969), nerfilin (Hirao et al., 1995), strepin (Ogura et al., 1985), thiolstatin (Murao et al., 1985; Kamiyama et al., 1994), tyropeptin (Momose & Watanabe, 2017), and tyrostatin (Oda et al., 1989); and (ii) those in which the N-terminal group binds to a ureido moiety that is attached to an amino acid through an amide linkage, such as antipain (Suda et al., 1972; Umezawa et al., 1972), chymostatin (Umezawa et al., 1970), elastatinal (Hamao Umezawa et al., 1973; Okura et al., 1975), GE20372 (Stefanelli et al., 1995), and the so-called microbial alkaline protease inhibitor or MAPI (Watanabe et al., 1979; Murao & Watanabe, 2014). The chemical configuration of SPAs, more notably in the latter ureido-containing class, allows alteration of the peptide chain, from N-terminal to C-terminal into C-terminal to C-terminal, making them dissimilar to ribosomally synthesized peptides.

Previous analysis of the genome of *Streptomyces lividans* 66 and the closely related organisms *S. coelicolor* M145 and *S. lividans* TK24 led to the detection and further prediction of an unprecedented peptide biosynthetic gene cluster (BGC) unique to *S. lividans* strain 66 (Cruz-Morales et al., 2013). The predicted biosynthetic system includes (i) an NRPS-like protein, lacking both canonical condensation (C) and thioesterase (TE) domains, but with one adenylation (A) domain, a peptidyl carrier protein (PCP) and a reductase (R) domain (*Sli0883* gene); (ii) a homolog of a leucyl/phenylalanyl (L/F) tRNA transferase (*Sli0884* gene), a tRNA-utilizing enzyme (tRUE) hitherto never implicated in natural products (NP) biosynthesis; and (iii) an *N*-acyltransferase (*Sli0885* gene). These enzymes are located within the SLP3 linear plasmid, a mobile genetic element of *S. lividans* absent from the industrial strain TK24 (Cruz-Morales et al., 2013). This prediction is congruent with the synthesis of an *N*-acylated leu-arginal dipeptide, which we called livipeptin, with predicted structural features similar to those present in leupeptin, thiolstatin, and bacithrocins, amongst other acetyl-leu-leu/arginal compounds. Thus, it was hypothesized that the product of this BGC, that is livipeptin, would have protease inhibitory activity.

Here, we characterize *S. lividans* 66 putative livipeptin's BGC following a combined approach, consisting of evolutionary genome mining, chromatographic bioactivity fractionation, and genetic experiments. Our results show that this unprecedented biosynthetic system directs the synthesis of a metabolite with protease inhibitory activity, yet very unstable, hampering chemical characterization and thus structural elucidation of livipeptin remains to be accomplished. Genetic and semisynthetic chemical experiments in the presence of RNAse did nevertheless confirm the involvement of an L/F transferase, previously linked to protein tagging during proteolysis (Varshavsky, 2011), in NP biosynthesis. Our findings represent a valuable set of unprecedented molecular beacons that can facilitate the genome mining of NP (Baltz, 2021) with protease inhibitory activity.

## Results and Discussion

### Evolutionary Genome Mining of Actinobacterial L/F Transferases Identifies tRUEs Involved in NP Biosynthesis

The presence of an L/F transferase homolog in the presumed livipeptin BGC, which includes NRPS-like genes, suggested that this enzyme family could have been recruited to synthesize NPs. This possibility encouraged us to mine for L/F transferases in the context of NP biosynthesis, emphasizing Actinobacteria, as close homologs of *Sli0884* linked to *Sli0883* could not be found beyond this phylum. Thus, combined EvoMining (Sélem-Mojica et al., 2019) and CORASON (Navarro-Muñoz et al., 2020) phylogenomic analyses of actinobacterial L/F transferases allowed us to identify 137 L/F transferase homologs within 1246 good-quality and well-annotated actinobacterial genomes. Remarkably, even though L/F transferases are known to play a central role as housekeeping enzymes involved in proteolytic metabolism (Mogk et al., 2007), only 11% of the actinobacterial genomes investigated include a homolog of this gene, suggesting that these organisms may have alternative proteolytic tagging strategies, such as the SsrA (tmRNA) tagging system (Braud et al., 2006).

**Fig. 2.**
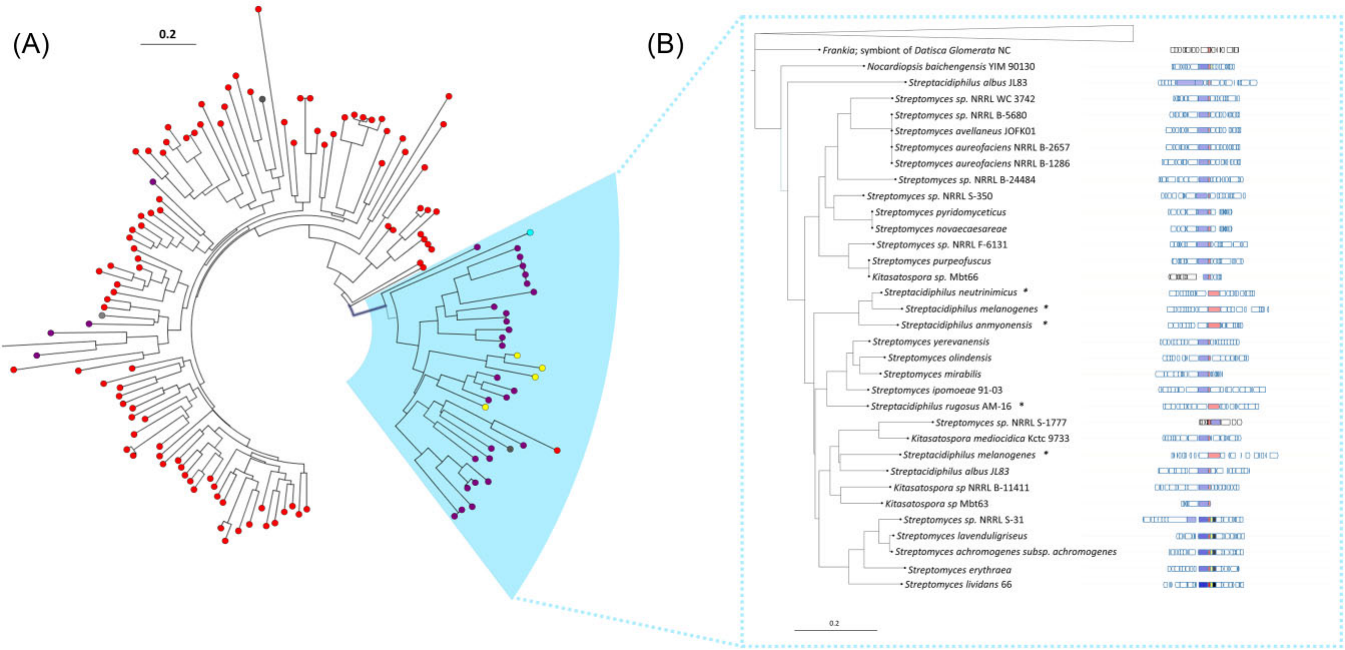
Combined EvoMining and CORASON analysis of L/F transferases in Actinobacteria. (A) EvoMining. Different metabolic roles or fates for the L/F transferase homologs were predicted: central metabolism (red), transition enzyme (purple), NP biosynthesis (turquoise), expanded enzymes (gray), and protein fusions with C-minus NRPS-like genes (yellow). The so-called small *Streptomyces* conserved clade is highlighted in blue and analyzed in (B) CORASON. The large non-conserved clade is used as the root of the small *Streptomyces* conserved clade, which conserves gene neighborhood. L/F transferase (red), C-minus NRPS-like (blue), *N*-acyltransferase (yellow), *O*-methyltransferase (black), and butyryl-CoA dehydrogenase (green). An asterisk (*) and pink are used to indicate protein fusions between the C-minus NRPS-like protein and L/F transferases.

The EvoMining and CORASON output revealed two discrete clades, consistent with one function possibly devoted to housekeeping metabolism and a second function predicted to be involved in NP biosynthesis. The large branch includes 104 enzymes from diverse Actinobacteria species without a conserved gene neighborhood (Supplementary Figure S1), supporting a single-enzyme housekeeping biochemical function. The second clade contains 33 entries and shows a more significant degree of synteny. These enzymes are mainly from the genus *Streptomyces* but also from *Nocardiopsis* (1), *Kitasatospora* (3), *Streptacidiphilus* (6), and *Actinopolyspora* (1), with a *Frankia* non-conserved entry at its root (Fig. 2). This so-called small *Streptomyces* conserved clade consists of L/F transferases probably recruited into NP biosynthesis, as revealed by EvoMining and the presence of other known NP enzymes. Interestingly, our analyses also revealed potentially translational fusions between the L/F transferase tRUE and the C-minus NRPS-like genes (shown with an asterisk, Fig. 2B). As in *S. lividans*, the latter was consistently found to encode for an A domain predicted to have specificity toward an arginine residue, plus a PCP and an R domain.

**Fig. 3.**
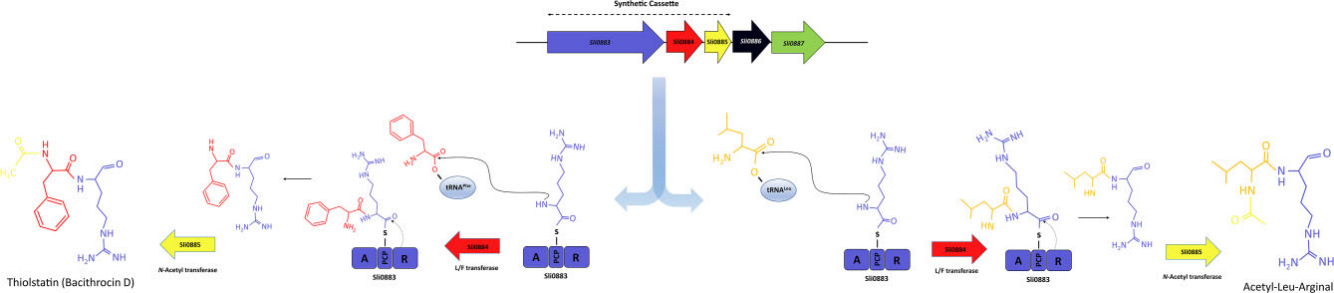
Biosynthetic proposal for livipeptin biosynthetic pathway. Dotted lines above the BGC indicate the region included in the synthetic cassette for the heterologous experiments in *E. coli*. Refer to the text for details about livipeptin biosynthetic proposal.

The domain organization confirmed above resembles that of a canonical NRPS involved in synthesizing other protease inhibitors (Kaysser, 2019), but with the L/F transferase replacing the expected C domain needed to form an amide bound. The R domain could be functionally equivalent to that in flavopeptins and antipain, whose biosynthesis involves an NRPS with an R domain responsible for the reductive release of the nascent peptide (Chen et al., 2013; Maxson et al., 2016). Within the small *Streptomyces* sub-clade, the livipeptin BGC branch includes hits from four other *Streptomyces* species and one from *Actinopolyspora erythreae* JPMV. Yet, despite these species being from different genera, their BGCs share high sequence similarity and conserved gene order. This includes two other biosynthetically potential elements: an *O*-methyltransferase and a butyryl-CoA dehydrogenase, that is *Sli0886* and *Sli0887* in *S. lividans*. Altogether, these observations support our previous prediction related to the synthesis of the putative protease inhibitor livipeptin in *S. lividans* 66 (Cruz-Morales et al., 2013), including both leucine and phenylalanine as alternative biosynthetic precursors (Fig. 3). Furthermore, this prediction is consistent with an acetyl-leu-arginal similar to previously isolated SPAs, namely, the bacithrocins and thiolstatin isolated from *Brevibacillus laterosporus* (Kamiyama et al., 1994).

Based on the abovementioned observations, we propose a biosynthetic logic involving a small assembly line that catalyzes amide bond formation between arginine, encoded by the A domain of the NRPS-like gene, and phenylalanine or leucine residues provided by their cognate aminoacyl-tRNAs as substrates of the divergent L/F transferase tRUE. A reductive release mechanism leading to the aldehyde moiety of livipeptin would also be part of this pathway. The resulting dipeptide aldehyde may undergo acetylation catalyzed by an *N*-acetyltransferase. Our prediction implies that these three enzymes may represent the minimal biosynthetic core necessary to synthesize a metabolite with protease inhibitory activity. However, additional methylation (*Sli0886*) and further dehydrogenation (*Sli0887*) could also be in place (Fig. 3). This biosynthetic proposal contrasts with previous data for leupeptin in pathogenic gammaproteobacteria (MIBiG BGC00021354, Li et al., 2020), which also differs from the NRPS-mediated synthesis of antipain (MIBiG BGC0002051), chymostatin, leupeptin, elastatinal, MAPI (Maxson et al., 2016), as well as flavopeptins (Chen et al., 2013), produced all by *Streptomyces* spp. These observations suggest convergent evolution leading to similar chemical scaffolds sustaining protease inhibitory activity via aldehydes, with physiological relevance as previously suggested for *Streptomyces* (Chater et al., 2010) and shown for other bacteria (Li et al., 2020).

### The *S. lividans* 66 Presumed Livipeptin BGC Encodes Protease Inhibitory Activity

To identify the livipeptin metabolite(s) potentially produced by *Sli0883-5*, an *S. lividans* 66 mutant was constructed, that is *S. lividans* ΔLvp (*Sli0883-5* minus), and the presumed protease inhibitory activity was followed in the *S. lividans* 66 wildtype strain during bioactivity fractionation of aqueous extracts of cultures grown on inducing conditions (Cruz-Morales et al., 2013). Despite all our optimization efforts, however, we failed to obtain suitable conditions leading to unambiguous metabolite identification and purification in the active fraction of wildtype *S. lividans* strain 66, which was absent from the ΔLvp mutant at the relevant retention time (RT 4.5 min, Table 1). Previously, similar difficulties were encountered during chemical characterization of antipain and analogs, which were attributed by the occurrence of multiple stereoisomers, their rapid conversion, and the lack of easy-to-follow chromophores in the structure of this compound (Maxson et al., 2016). So, predicted mass of livipeptin could not be confirmed (Supplementary Figure S2). Moreover, degradation of the 4.5 min chromatographic peak was seemingly apparent, as revealed by decay of the protease inhibitory activity detected during the fractionation and the size of the High Performance Liquid Chromatography (HPLC) peak. These circumstances, which contrast with leupeptin produced by *Streptomyces roseus* ATCC 31245 in our hands (RT 28.5 min for an active and stable fraction, see below), unfortunately hampered our ability to chemically characterize livipeptin.

**Table 1.** Protease Inhibitory Activity of Native and Semisynthetic Livipeptin

				T. I. A.^b^	P. I. A. ^b^
Organism	Genotype	Metabolite	RT^a^ (min)	(%)	(%)
*S. lividans* 66	WT	Livipeptin	4.5	10.5 ± 1.1	3.5 ± 0.4
*S. lividans* ΔLvp	WT *Sli0883-5*^−^		NI	88 ± 4.1	85.4 ± 1.6
*E. coli* pAsk	C41 pAsk		NI	87.5 ± 10.9	100 ± 6.4
	(empty vector)				
*E. coli* pLvp	*E. coli* C41 pLvp (*Sli0883-84-85*)	Livipeptin	4.5	12.7 ± 0.6	11.3 ± 1.8
*S. roseus* ATCC 31 245	wild type (WT)	Leupeptin	28.5	9.6 ± 0.7^d^	
**Reaction Mix**	**Genotype**			**T. I. A.** ^b^	**P. I. A.** ^b^
**(*E. coli* c. f. e.)** ^c^	**(plasmids)** ^d^	**Metabolite**	**RT** ^a^ **(min)**	**(%)**	**(%)**

pAsk	Empty vector	NI	NI	87.5 ± 10.9	100 ± 6.4
pLvp	*Sli0883-4-5*	Livipeptin	4.3	12.7 ± 0.6	11.3 ± 1.8
+ RNase		NI	NI	98.7 ± 6.9	99.8 ± 8.2
Sli3, pSli4, pSli5	*Sli0883, Sli0884, Sli0885*	Livipeptin	4.3	60.5 ± 5.5	16.4 ± 0.4
+ RNase		NI	NI	93.9 ± 7.2	93.2 ± 4.4
Sli3, Sli4-5	*Sli0883, Sli0884-5*	Livipeptin	4.3	62.5 ± 2.5	11 ± 0.5
+ RNase		NI	NI	100 ± 4.6	89.9 ± 7.3
Sli3-4, Sli5	*Sli0883-4, Sli0885*	Livipeptin	4.3	66.8 ± 7.7	12 ± 0.4
+ RNase		NI	NI	98.9 ± 4.7	100 ± 2.1
Sli3-4	*Sli0883-4*	NI	NI	99.2 ± 9.1	98.1 ± 7.9
Sli4-5	*Sli0884-5*	NI	NI	90.4 ± 9.0	100 ± 4.7
Sli3	*Sli0883*	NI	NI	100 ± 8.2	100 ± 3.1
Sli4	*Sli0884*	NI	NI	100 ± 5.9	100 ± 4.4
Sli5	*Sli0885*	NI	NI	92.5 ± 5.4	100 ± 4.6

^a^HPLC retention time (RT). NI, not identified (see Fig. 4 and its associated Supplementary Figure S2 for spectral analysis).

^b^T.I.A. and P.I.A., trypsin and papain inhibitory activity, respectively, recorded as activity curves for 20 min and reported as a percentage of the activity found for leupeptin and papain, respectively (see also Fig. 4A).

^c^The reaction mixtures were prepared with *E. coli* cell-free extracts (c. f. e.) with or without the addition of RNase.

^d^Genotype provided is for plasmids harboring the *S. lividans* 66 genes *Sli0883-5*. NI, activity could not be identified. As in Fig. 4, *Sli0883-5* genes are shown only with their final digit, that is 3, 4, and 5. A comma (,) and a hyphen (-) are used to denote expressions *in trans* or *in cis*, respectively. Data were generated after three independent experiments, leading to standard deviations measured as RFUs. ^d^Reported activity is from commercial leupeptin of microbial source with ≥90% purity (Sigma, No. L2884).

We therefore shifted our focus on the characterization of the protease inhibitory activity of livipeptin and the genes involved in encoding this activity. For these experiments, we used commercial leupeptin and antipain as positive controls against trypsin and papain, respectively. For leupeptin, we used a commercial standard, which is produced after bacterial fermentations, plus extracts of the leupeptin producer *S. roseus* ATCC 31245 (Table 1). For livipeptin, in addition to wildtype strain 66 grown on inducing conditions, we opted for an heterologous expression system consisting of synthetic *Sli0883-5* genes optimized for *Escherichia coli* codon usage, and the inducible pAsk vector (Adams et al., 2014). The resulting expression plasmid, termed pLvp, was used to transform *E. coli* C41, and the resulting strain, that is *E. coli* C41 Lvp, was cultivated in minimal medium. The conditions for these experiments allowed us to improve chromatographic comparative analyses of aqueous extracts, as they showed less residual metabolites than in the *S. lividans* native system. Yet, protease inhibitory activity decay still occurred too fast to attempt chemical characterization (Supplementary Figure S2). However, these data unequivocally establish a link between the *lvp* genes *Sli0883-5* and a putative SPA with protease inhibitory activity.

**Fig. 4.**
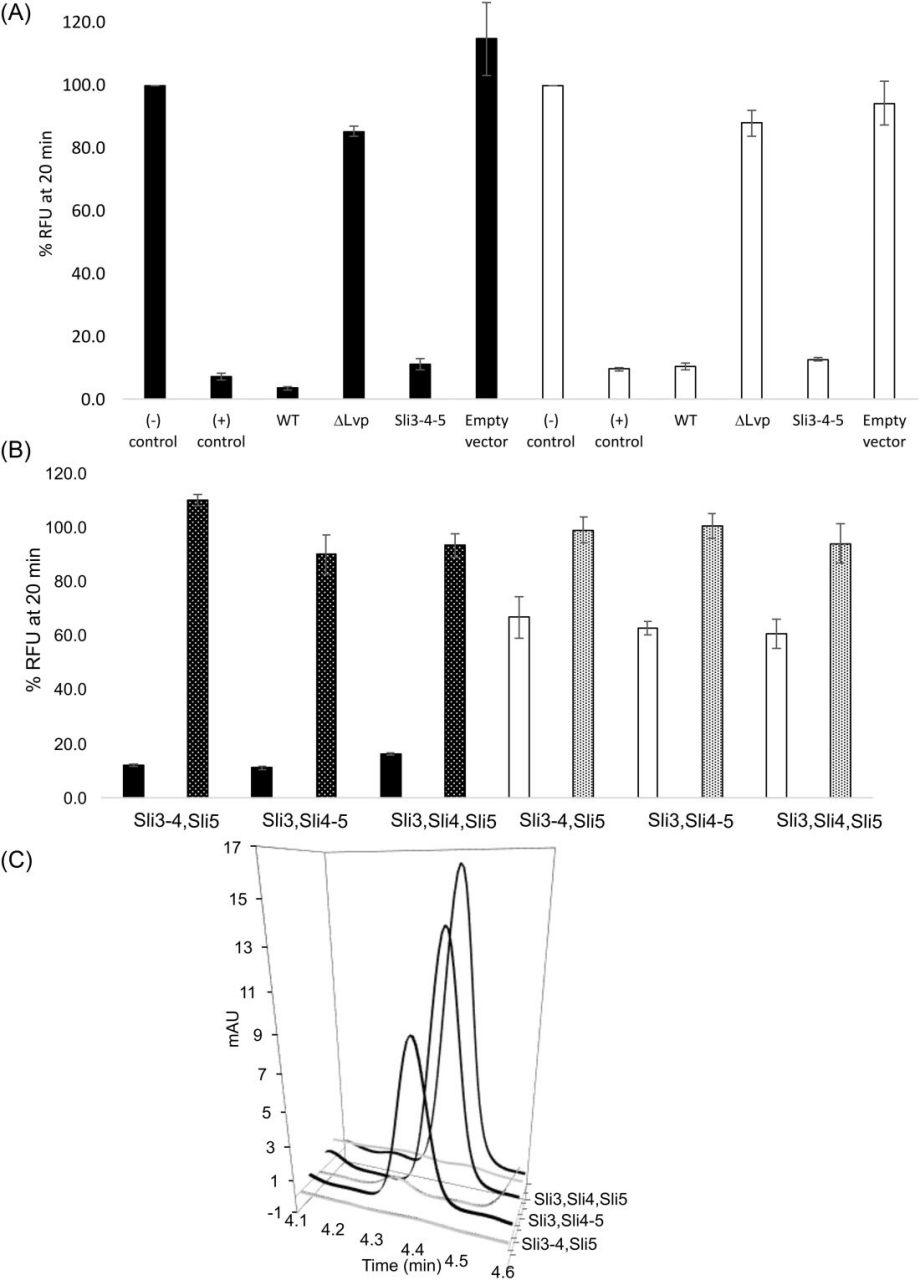
Papain and trypsin inhibitory activity of presumed livipeptin BGC enzymes. (A) HPLC fractions collected were analyzed in vitro using a fluorometric assay to demonstrate the proteolysis of the substrates as RFUs. The enzyme inhibition assays against papain (black bars) employed the chromogenic compound BApNA as substrate, and as positive control antipain for trypsin (white bars), the positive control was leupeptin. Results for both native (*S. lividans* WT and ΔLvp mutant) and heterologous (pLvp and empty vector) systems are shown. For the sake of clarity, *Sli0883-5* genes are shown only with their final digit, that is 3, 4, and 5. A comma (,) and a hyphen (-) are used to denote expressions in *trans* or in *cis*, respectively. The standard deviations shown were calculated from three independent experiments (Table 1). (B) Activity reconstitution using a synthetic biology approach of livipeptin BGC. Metabolites, proteases, and controls are in panel A (data not shown). Only the three three-gene reaction mixtures, irrespective of their genetic organization (expression in *trans* or in *cis*), showed protease inhibitory activity. Cell-free extracts with one- or two-genes constructs did not show inhibitory activity (Table 1). RNase (dotted bars) eliminates trypsin and papain inhibitory activity in around 87% and 37%, respectively. (C) Reconstitution of livipeptin biosynthesis used for experiments of panel B, showing a tRNA catalytic dependency (first trace of each dataset).

Previous data suggest that thiolstatin, whose structure would be the closest to that predicted for livipeptin, has weak inhibitory activity against serine proteases, such as trypsin, but potent proteolytic inhibitory activity toward cysteine proteases, such as papain. These observations explain the use of the prefix “thiol” in its name (Murao et al., 1985). Unfortunately, thiolstatin or bacithrocins standards are not available, making comparison of these metabolites with semi-purified livipeptin impossible. Thus, we focused on HPLC-purified fractions from *S. lividans* 66 and *E. coli* C41 pLvp cultures to establish some comparisons (Table 1 and Fig. 4). Although strong inhibitory activity equivalent to leupeptin and antipain could be found for the HPLC semi-purified metabolite(s) from the two experimental sources investigated (even when they showed slightly different RTs, 4.5 and 4.3 min, respectively), the material obtained from the *E. coli* heterologous system showed less activity, especially toward papain. Interestingly, the extracts from *S. lividans* 66 have more metabolite diversity than those from the *E. coli* experiments. Similar to leupeptin (Aoyagi et al., 1969) and antipain (Maxson et al., 2016) produced under rich *Streptomyces* growth conditions, livipeptin could be produced and co-purified as a molecular cluster, leading to synergistic protease inhibitory activity. Commercial high-quality leupeptin isolated from *S. roseus* cultures indeed includes several related species, contrasting with the less active synthetic leupeptin (McConnell et al., 1990).

### Livipeptin Biosynthesis Involves a C-minus NRPS—tRUE Mediated Biosynthetic Logic

In addition to codon usage optimization for *E. coli* expression, the pLvp plasmid was designed such that each of the three *lvp* genes, that is *Sli0883-85*, could be excised and the plasmid circularized with a different-yet-compatible cohesive restriction enzyme, easing construction of expression plasmids with all possible combinations, including expression *in cis* and *in trans* (Supplementary Figure S3). This synthetic biology strategy was designed to allow us to evaluate the functional impact that co-expression of two enzymes could have, providing a sense of the predicted enzyme-enzyme interaction of the C-minus NRPS-like protein, the L/F-transferase, and the *N*-acetyl transferase. In addition to pLvp (pSli3-4-5), five different constructs were obtained: three single-gene plasmids (pSli3, pSli4, and pSli5) and two double-gene plasmids (pSli3-4 and pSli4-5). The resulting plasmids were confirmed after DNA re-sequencing and used in assays consisting of mixtures of different cell-free extracts expressing the plasmids (Fig. 4B).

These experiments showed that only the three-gene combinations, after independent expression *in trans*, that is from different plasmids, or co-expression *in cis*, that is from the same plasmid, yielded the presumed livipeptin, as judged by detection of a fraction with protease inhibitory activity. We also noted that this fraction did not only show fewer metabolites than the active fraction from *S. lividans* 66 but eluted a bit earlier, around 4.3 min, as noticed previously. Protease inhibitory activity was similar between the *S. lividans* native system (Fig. 4A) and the heterologous *E. coli* system (Fig. 4B) against trypsin and, to a lesser extent, papain, whereas neither of the combinations with only two or one genes rendered active fractions. Thus, these results further establish the *Sli0883-5* genes as the minimal biosynthetic core of the livipeptin BGC encoding expression of protease inhibitory activity in *S. lividans* 66.

This experimental layout also allowed us to test the hypothesized aminoacyl-tRNA dependency of the L/F transferase tRUE, following the same approach adopted during the characterization of the tRNA-dependent lantibiotic dehydratase NisB involved in nisin biosynthesis (Ortega et al., 2014). For these experiments, cell-free extracts of cultures expressing the gene combinations containing the entire cluster were pre-incubated with RNase prior to generating the reaction mixture. As shown in Table 1 and Fig. 4B, bioactivity and HPLC analysis shows that addition of RNase suppresses protease inhibitory activity, concomitant with disappearance of the 4.3 min peak (Fig. 4C), which can be explained by the degradation of the aminoacyl-tRNA substrate. These findings contribute to the growing evidence highlighting the involvement of central metabolic tRUEs in natural product biosynthesis (Hong et al., 2004; Garg et al., 2008; Zhang et al., 2011; Belin et al., 2012; Bougioukou et al., 2013; Ortega et al., 2014). However, the remarkable aspect of the livipeptin biosynthetic pathway lies in its small size compared to other BGCs. This is made possible by the catalytic versatility of the tRNA L/F-transferase, which is predicted to substitute the function of the C domain of the NRPS-like protein during the biosynthesis of livipeptin.

To the best of our knowledge, this is the first time such a role has been described. The combinatorial potential provided by the interaction between A domains and tRUEs warrants further investigation in many ways. For instance, we envisage exploitation of this discovery for uncapping novel NPs diversity through genome mining efforts and for developing synthetic biology approaches targeting proteolysis in different settings (Hines et al., 2008), such as within the recent COVID-19 pandemic crisis (Fu et al., 2021).

## Materials and Methods

### Phylogenomics and Genome Mining of Natural Products

Enzymes from four different microorganisms, namely, *S. lividans, Rubrobacter xylanophilus, Frankia alni*, and *Propionibacterium acnes*, together with the L/F transferase homolog from the livipeptin BGC, were used as query sequences to retrieve homologs from an in-house 1246 actinobacterial genome database. From this search, 140 homologous sequences were obtained and used to look for known NPs biosynthetic pathways recruitments in the MIBiG database (Terlouw et al., 2023). The presumed BGC for livipeptin was curated and previously deposited under the accession number BGC0001168. The 140 L/F transferase sequences were aligned and trimmed prior to constructing the phylogenetic tree, which was done with Seaview BioNeighbor Joining Poisson software in the standard calibration mode. EvoMining (Cruz-Morales et al., 2016; Sélem-Mojica et al., 2019) and CORASON (Navarro-Muñoz et al., 2020) algorithms were used as previously.

### Construction of the *S. Lividans* Mutant and *E. Coli* Overexpressing Strains

For the identification and characterization of livipeptin in *S. lividans* 66, a mutant deficient for the *Sli0883-5* genes was constructed. These genes were replaced by the apramycin resistance cassette *aac(3)IV* marker in-frame within a pESAC13-A construct isolated from a genomic library constructed by Bio S&T (Québec, Canada). A 1.5 Kb region flanking the *Sli0883-5* genes replaced by the apramycin cassette was then amplified by polymerase chain reaction (PCR) and cloned into the plasmid pWHM3, which contains a thiostrepton resistance gene. pWHM3 is an unstable *Streptomyces* vector that is lost after some rounds of cultivation of the transformed strain without selection (Vara et al., 1989). Double crossovers after integration of the pWHM3 *Sli0883-5:: aac(3)IV* construct were screened after apramycin resistance (50 μg/mL) and thiostrepton (25 μ/mL) sensitivity. The genotype of several transformants was confirmed by PCR, leading to *S. lividans* 66 ΔLvp used for experimentation. The synthetic presumed livipeptin BGC optimized for *E. coli* codon usage was obtained from GeneScript (NJ, USA). The design of the genetic construction includes restriction sites flanking each gene, as follows: *Nde*I-*Sli0883*-*Eco*RI-*Sli0884*-*Xba*I-*Sli0885*-*Bgl*II-*Hind*III. Different combinations of these genes were cloned into pAsk (Adams et al., 2014), resulting in six different plasmids (Table 1 and Supplementary Figure S3). The leupeptin-producer *S. roseus* ATCC 31245 was purchased from the ATCC.

### Culture Conditions and Sample Preparation for Metabolites Analysis

Culture conditions for the induction of the presumed livipeptin BGC were as previously (Cruz-Morales et al., 2013). Briefly, 50 mL shake flask cultures were inoculated with approximately 1 × 10^6^ fresh spores of *S. lividans* 66. Cultures were incubated at 30°C for 48 h on R5 modified medium, without potassium phosphate, 0.2 g/L casaminoacids, and 200 mM MgCl_2_ (Kieser et al., 2000). The culture supernatants were collected for HPLC analysis. For heterologous livipeptin production, *E. coli* C41 Lvp strain was used to inoculate Luria broth (LB) cultures overnight and used as inoculum for 250 mL baffled flask with 50 mL of M9 medium (glucose 4 g/L added with L-arginine and L-phenylalanine 0.5 g/L each) and incubated at 30°C and 200 rpm. The cultures were started at 0.1 OD_600_ and induced with 20 ng/µL of anhydrotetracycline. The culture was maintained for 24 h, and the supernatant was collected for HPLC-MS analysis. For the production of leupeptin, *S. roseus* ATCC 31245 was grown on shake flask cultures containing the following media: glucose 3 g, NH_4_NO_3_ 0.5 g, MgSO4 (7H_2_O) 0.5 g, KCl 0.05 g, L-leucine 0.75 g, L-arginine 0.75 g, glycine 0.75 g, casaminoacids 0.1 g, yeast extract 0.4 g per liter. Cultures were incubated at 30°C for 48 h before supernatant analysis.

The cell-free extracts of *E. coli* C41 were obtained as previously (Kigawa et al., 2004) with some modifications. *E. coli* C41 pGroEL/GroES was inoculated in 50 mL of LB medium and grown at 37°C to an OD_600_ of 0.5 for induction with 20 ng/µL of anhydrotetracycline. After induction, the culture was grown at 20°C for 12 h. Cells were harvested (8000 g, 10 min, 4°C) and the pellet was washed three times by resuspension in S30 buffer (10 mM Tris-acetate buffer pH 8.2, 14 mM magnesium acetate, 60 mM potassium acetate, 1 mM dithiothreitol, 0.3 mM EDTA, 0.3 mM MgCl_2_) followed by centrifugation (8000 g, 10 min, 4°C). Cells were then resuspended in 1 mL of S30 buffer per gram of wet cells and lysed with a Sonicator. The cell lysate was centrifuged twice (35 000 g, 45 min, 4°C) and the supernatant was dialyzed four times against 50 volumes of S30 buffer (without dithiothreitol) using Amycon Ultra-15 (Merck Millipore) tubes for dialysis with a molecular mass cutoff of 10 kDa. The cell extract was then centrifuged (1000 g, 10 min, 4°C) and the supernatant was frozen and stored in 1 mL samples at −80°C for future use.

### HPLC metabolite profiling and fractionation

Supernatant of cultures and in vitro reaction mixtures were evaporated to dryness. The dry residues were dissolved in a 0.1 volume of HPLC grade H_2_O and injected into a C18 Discovery 504 955 Supelco column with a particle size of 5 μm connected to an HPLC-Agilent 1200 equipped with a diode array detector and a fraction collector. The mobile phase comprised a binary system of eluent A, H_2_O, and eluent B, 100% MeOH. The run consisted of H_2_O/MeOH gradient (0–5 min: 0% B; 5–35 min: 10% B; 35–45 min: 100%). Differential peaks between the wildtype and mutant strain (or empty vector) were detected by monitoring absorbance at a wavelength of 280 nm, and the selected fractions were collected for bioactivity assays.

### Protease Inhibitory Enzyme Assays

HPLC fractions collected were analyzed in vitro using a fluorometric assay with excitation at 340 nm and emission at 420 nm. The chromogenic compound Nα-Benzoyl-DL-arginine 4-nitroanilide hydrochloride (BApNA, Sigma-Aldrich) was used as the substrate for trypsin and papain (Sigma-Aldrich T1005 and P3375, respectively), and leupeptin standard (Sigma, No. L2884) as a positive control. HPLC peaks collected were dried in a vacufuge and then resuspended in Milli-Q water. The assay mixture for trypsin inhibition contained Tris-HCl (0.1 M pH 8) as reaction buffer, trypsin (0.05 mg/mL), substrate BApNA (0.1 mg/mL), leupeptin (0.001 mg/mL as positive control) or collected peaks (50 μL). Papain inhibition assay was done on a phosphate reaction buffer with DTT (100 mM), EDTA (60 mM), papain (0.01 mg/mL), antipain (0.01 mg/mL as positive control) or collected peaks (50 μL). The following reaction mixture in a final volume of 200 μL was used for the in vitro protease assays: *E. coli* cell-free extracts or HPLC fraction (10 μL), HEPES pH 7.5 (100 mM) dithiothreitol (1 mM), L-lysine (10 mM), L-leucine (10 mM), L-Arginine (10 mM), L-Phenylalanine (10 mM), MgCl_2_ (10 mM), KCl (10 mM), ATP (5 mM), and NADPH (5 mM). The assay was incubated at 30°C for 5 h, and centrifugated to remove insoluble material (35 000 g, 5 min, 25°C). In addition, the cell-free extract (10 μL) was treated with RNase in the presence of CaCl_2_ (100 μM). The activity was calculated in percentage using as 100% the relative fluorescence units (RFUs) of the proteases without inhibitors at the end of the reaction (20 min). The slope of the curve (initial rate) of the time progress of the reaction in each experiment was also calculated by triplicate.

## Supplementary Material

kuad021_Supplemental_Figures
