# Supplementary figures and images for: Streptomyces lividans 66 produces a protease inhibitor via a tRNA-utilizing enzyme interacting with a C-minus NRPS
Source: J Ind Microbiol Biotechnol. 2023 Sep 5;50(1):kuad021. doi: 10.1093/jimb/kuad021 (PMC10548850; doi:10.1093/jimb/kuad021)

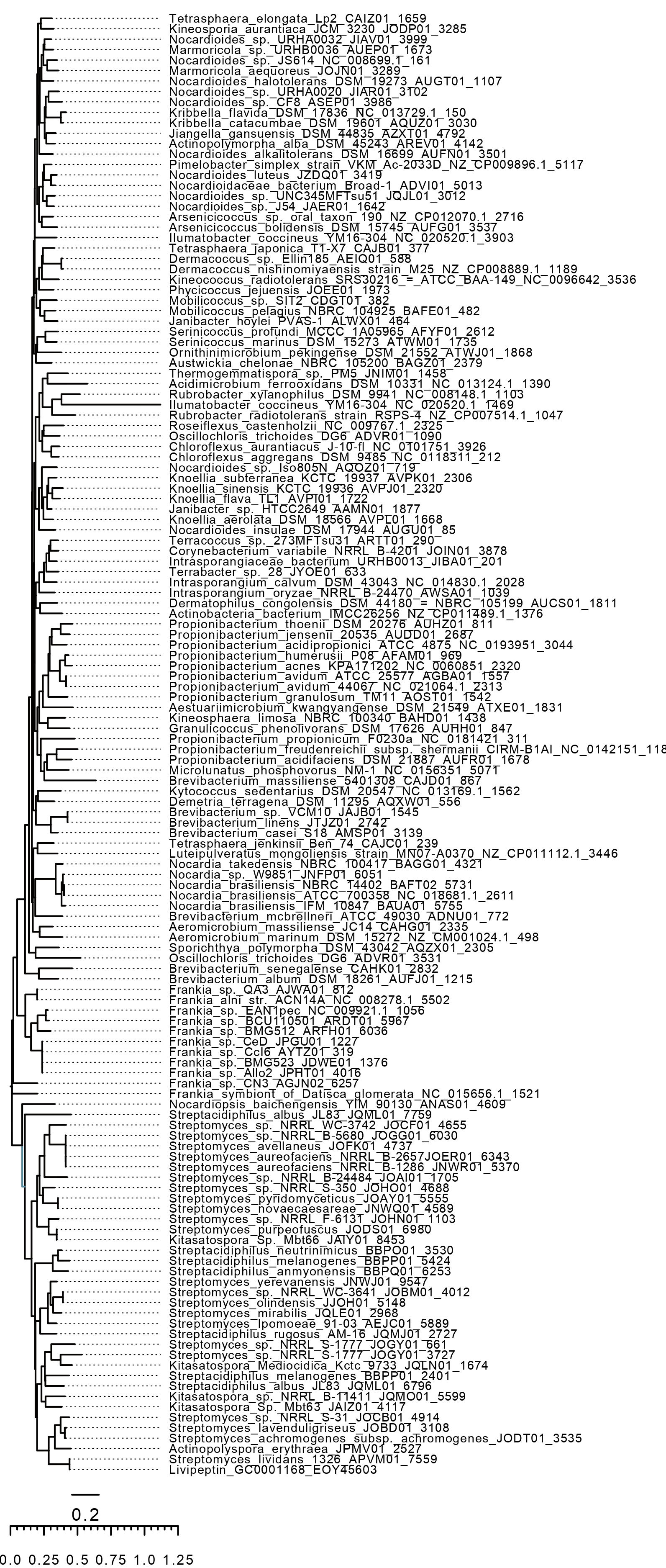

Supplement: kuad021_Supplemental_Figures [file kuad021_supplemental_figures.zip › Figure_S1_Lvp.jpg]

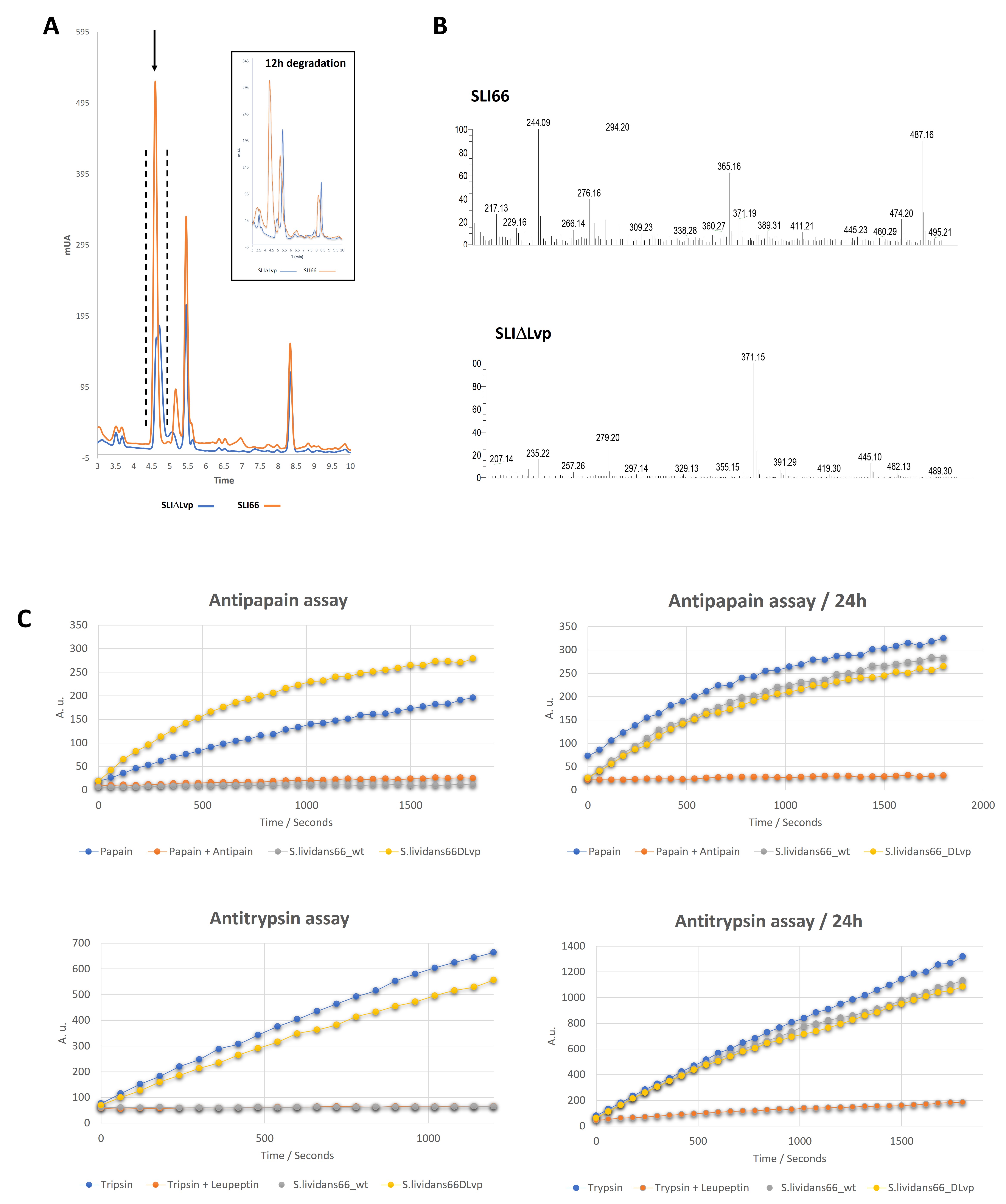

Supplement: kuad021_Supplemental_Figures [file kuad021_supplemental_figures.zip › Figure_S2_Lvp.jpg]

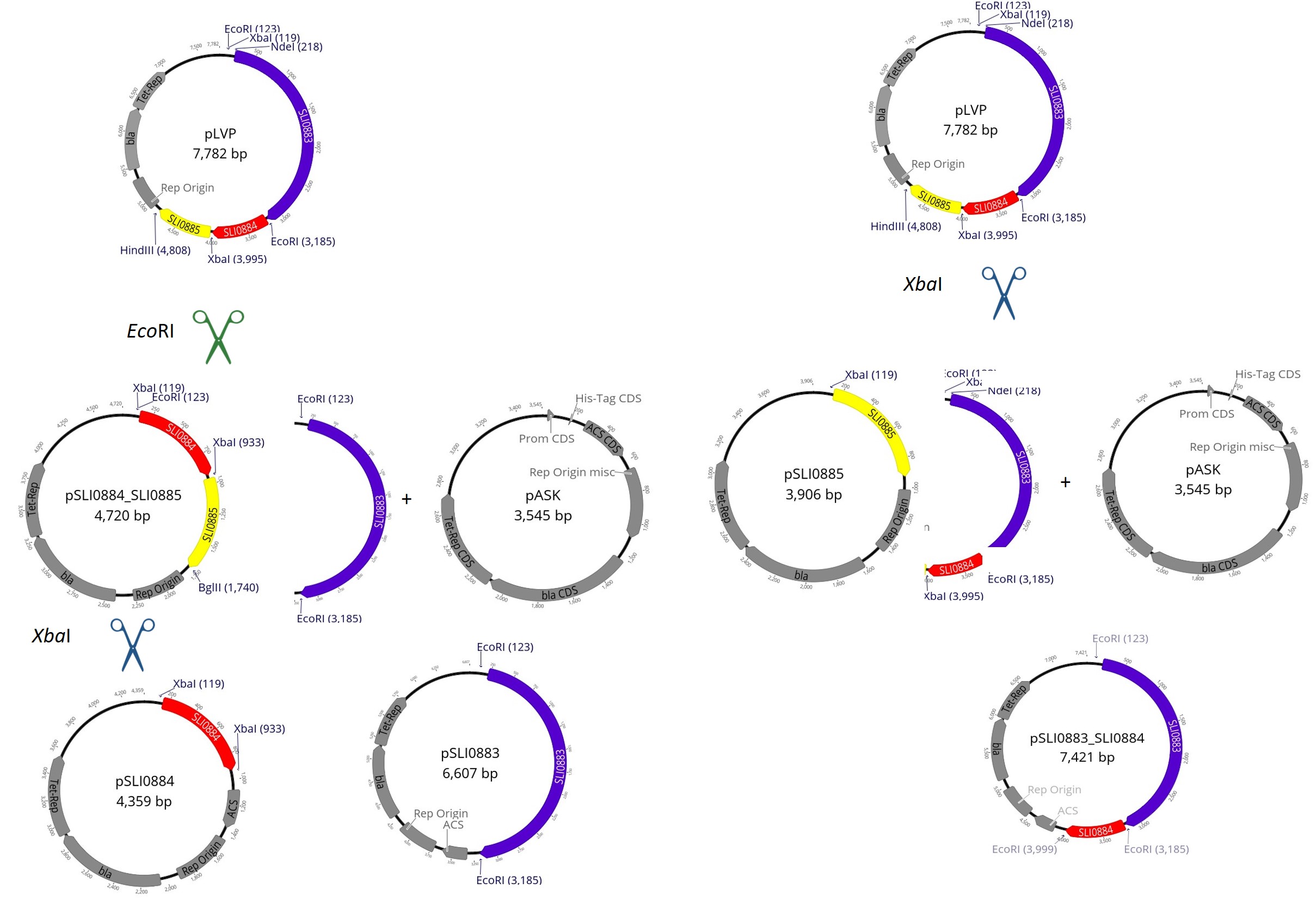

Supplement: kuad021_Supplemental_Figures [file kuad021_supplemental_figures.zip › Figure_S3_Lvp.jpg]
